# AI model predicts patient outcomes from surgical gestures and provides insights into explainability

**DOI:** 10.1038/s44484-025-00006-y

**Published:** 2026-05-04

**Authors:** John R. Heard, Atharva Deo, Umar Ghaffar, Runzhuo Ma, Cherine Yang, Ujjwal Pasupulety, Jasmine Lin, Carter M. Prentice, Hyung L. Kim, Melissa Assel, Christian Wagner, Geoffrey A. Sonn, Alvin C. Goh, Andrew Vickers, Jim C. Hu, Andrew J. Hung

**Affiliations:** 1 Department of Urology, Cedars-Sinai Medical Center, Los Angeles, CA USA; 2 Department of Urology, New York Presbyterian Hospital, Weill Cornell Medical Center, New York, NY USA; 3 Department of Surgery, University of California, Los Angeles, CA USA; 4 Department of Epidemiology and Biostatistics, Memorial Sloan Cancer Center, New York, NY USA; 5 Department of Urology, Pediatric Urology and Urologic Oncology, St. Antonius-Hospital, Gronau, Germany; 6 Department of Urology, Stanford University Medical Center, Stanford, CA USA; 7 Department of Urology, Memorial Sloan Cancer Center, New York, NY USA

**Keywords:** Diseases, Medical research, Urology

## Abstract

Effective surgical training requires relatively immediate feedback as to outcomes. This makes surgical learning problematic as some surgical outcomes may take months or years to become apparent. The sequence of surgical gestures, the smallest discrete actions of surgery, during the nerve-sparing step of robot-assisted radical prostatectomy has been used to predict 1-year erectile function (EF) outcomes after surgery. To improve this prediction and extract clinically meaningful insights, we describe the addition of anatomic and functional context to surgical gestures. We analyzed surgical video of 147 patients at 5 surgical centers undergoing robotic-assisted radical prostatectomy. The addition of anatomic and functional characterization to surgical gestures improved model prediction of post-operative EF from 0.78 [95%CI: 0.60, 0.92] to 0.85 [95%CI: 0.66, 0.96]. Aggregated attention weight analysis identified novel gesture, anatomy, and function combinations contributing most to EF outcomes. The identification of these critical gestures provides a starting point for more data-driven training in clinical practice.

## Introduction

Surgical performance improvement requires relatively immediate feedback on outcomes. This is problematic for surgeons, as patient outcomes can take months to years after the operation to become apparent. As a prime example, erectile function (EF) recovery after robotic-assisted radical prostatectomy (RARP) may not manifest until at least one year after surgery^1,2^. To provide immediate actionable feedback, quantifiable metrics of surgical performance linked to surgical outcomes are needed.

Surgical gestures, the smallest discrete actions during a surgery such as *cut, spread*, or *coagulate*, are one such performance metric. The ratios of different gesture usage have been associated with surgeon experience, surgical skills, and patient outcomes across multiple surgical specialties^3–5^. This type of traditional biostatistical analysis, however, does not account for the temporal relationship between gestures during the course of surgery. To do so, our group previously used the *sequence* of surgical gestures as input for a machine learning (ML) model to predict EF outcomes one year after RARP^6^.

Although specific gestures or sequences of gestures have been shown to predict patient outcomes, whether by ML or traditional biostatistical methods, it is still not clear why these actions lead to better outcomes^4,7^. Currently, these associations do not provide the level of insight needed to infer causality or change surgeons’ practices. Additional insight into the effects of these gestures on patient anatomy, and what the gestures are intended to achieve, is needed in order to accurately assess why certain gestures are effective while others are harmful. Knowledge about the “where” (tissue anatomy) and “why” (function) of the gestures most important for surgical outcomes may provide greater insight into the mechanism of their effect and allow us to derive causal conclusions that surgeons can use to improve their surgical performance.

**Fig. 1:**
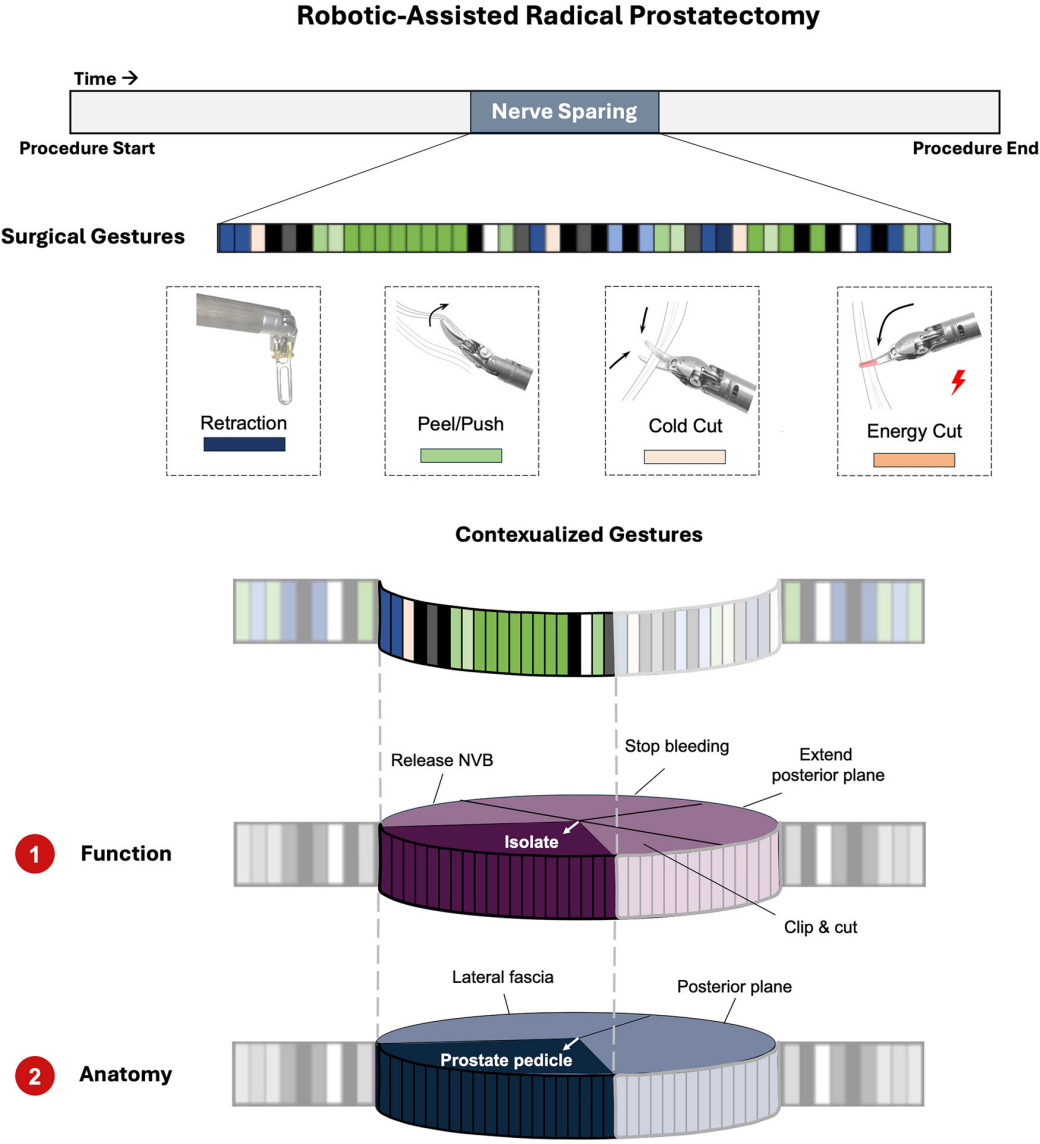
Schematic of contextualized gestures during the nerve sparing step of the robotic-assisted radical prostatectomy.

In this study we sought to improve the predictive ability and explainability of ML models for surgical gestures from our prior work by adding information about the anatomy and function to each gesture. In this new approach, gesture, anatomy, and function are combined to generate *contextualized gestures* (CGs) (Fig. 1), which we annotate from surgical videos of RARPs to predict patient EF outcomes.

## Results

### Study cohort and outcomes

After prospective recruitment, 147 patients fulfilled the eligibility criteria. These patients’ RARPs were performed by 26 surgeons at 5 surgical centers in 2 countries. The primary outcome of intact EF at one-year post-RARP was achieved by 35% (51/147) of patients. One-year EF was associated with age (*p* < 0.001), pre-op EF (*p* = 0.005), prostate volume (*p* = 0.013), and postoperative Gleason score (*p* = 0.025) (Supplemental Table 1). The median prior RARP caseload per surgeon was 2000 cases (IQR 653–2000). Prior caseload was not associated with one-year postoperative EF (*p* = 0.7).

### Gestures with anatomic and functional context

**Fig. 2:**
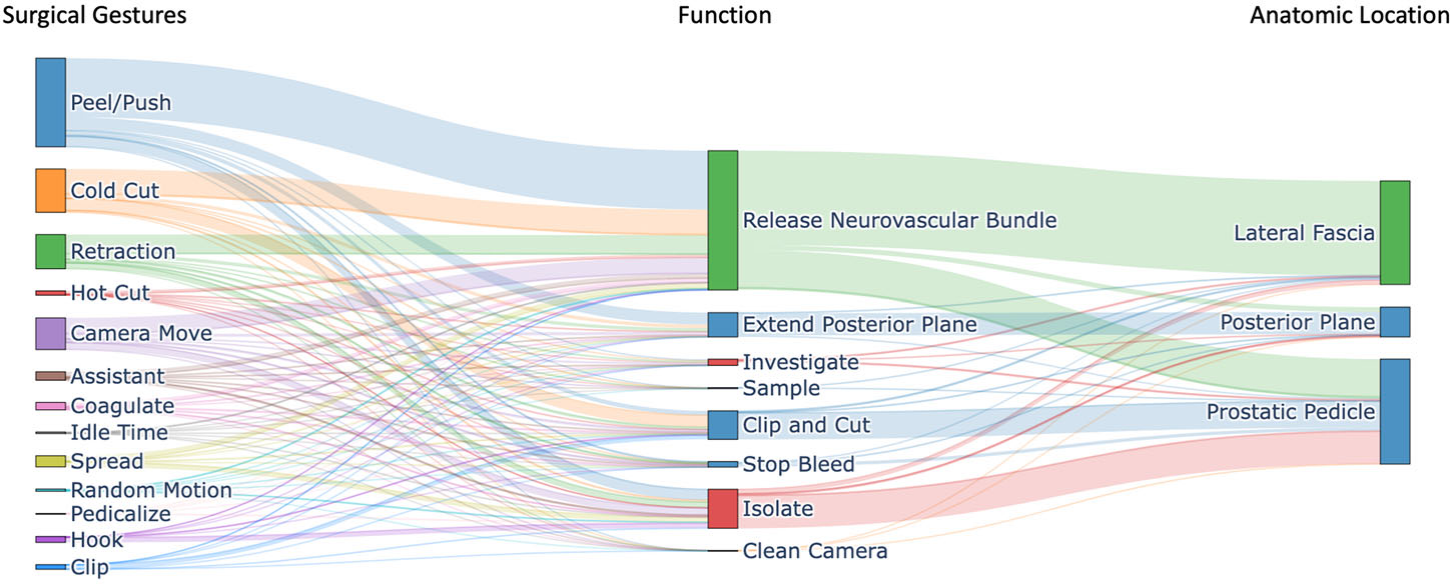
Sankey diagram depicting the relationship between surgical gestures, function and anatomical location.

The total number of surgical gestures annotated was 75,824, with a median of 446 gestures per nerve-sparing step (IQR 435–937). The median uses of each gesture and their proportion of total gestures can be found in Supplemental Table 2. Also reported are the median number and proportion of gestures used for each and in each anatomic location. This is depicted in Fig. 2. Briefly, the most frequently used gestures were *peel/push* (median 219 gestures/case (36%), IQR 111–142) and *cold cut* (103 gestures/case (17%), IQR 58-143). The function and anatomic location with the most gestures were *release neurovascular bundle* (322 gestures/case (54%), IQR 198–532) and *prostatic pedicle* (248 gestures/case (44%), 139–373). The most frequent combination of gesture, function, and anatomic location, herein referred to as a *contextualized* gesture (GC), was *peel/push* (gesture) for *release neurovascular bundle* (function) at the *lateral fascia* (anatomic location).

### Model performance with addition of anatomic and functional context

To assess if the anatomic location and function of gestures add clinically useful information, we evaluated their effects on the predictive performance of the transformer model for EF outcomes after RARP (Table 1). The AUC of the model using only gesture sequence alone was 0.78 (95%CI: 0.59, 0.93). The addition of anatomic location to gesture sequence increased the AUC to 0.80 (95%CI: 0.62, 0.94). The addition of both function and anatomic location to the gesture sequence yielded an AUC of 0.85 (95%CI: 0.71, 0.96). When the patient features of prostate volume, patient age, body mass index, post-op Gleason score, PSA, and prior surgeon caseload were added to the gesture sequence, function, and anatomy the AUC remained at 0.85 (95%CI: 0.75, 0.95).

**Table 1:** Transformer model predictive performance for erectile function recovery by input data

Included Data in Model				Model Performance	
Gesture Sequence	Anatomic Location	Function	Clinical Features	AUC (95% CI)	Δ AUC
●				0.78 [95%CI: 0.59, 0.93]	ref.
●	●			0.80 [95%CI: 0.62, 0.94]	0.025
●	●	●		0.85 [95%CI: 0.71, 0.96]	0.075
●	●	●	●	0.85 [95%CI: 0.75, 0.95]	0.075

Delta AUC based on comparison to reference. Clinical Features include: prostate volume, age, body mass index, post-op Gleason score, PSA and prior surgeon caseload.

### Explainability of model performance with contextualized gestures

**Fig. 3:**
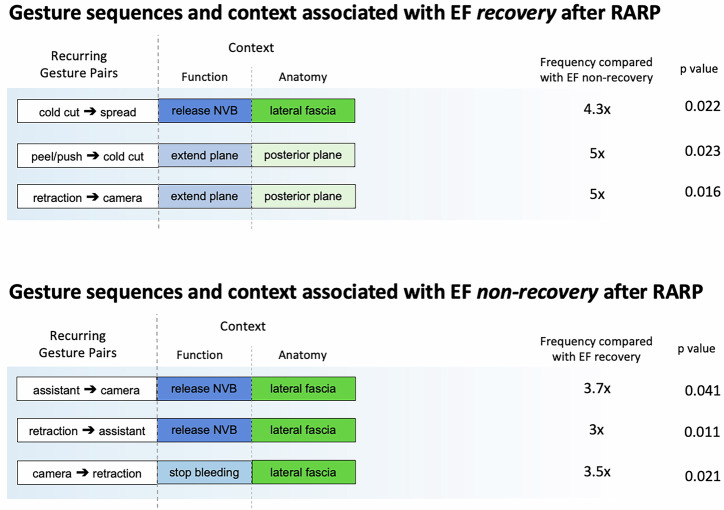
Sequential contextualized gestures most associated with EF outcomes and their frequencies.

Aggregated attention weight analysis yielded the single CGs and two sequential CGs contributing most to one year EF outcomes and their relative frequencies (Fig. 3). The most important sequential CGs associated with EF recovery was the transition from *cut* (surgical gesture) to *spread* for *release NVB* (function) at the *lateral fascia* (anatomy). The most important sequential CGs associated with EF non-recovery was the transition from *assistant move* (gesture) to *camera move* for *release NVB* (function) at the *lateral fascia* (anatomy).

## Discussion

Here we report that the addition of both anatomic location and function to surgical gestures each improves the ability of a transformer model to predict patient outcomes after surgery. Additionally, we identified the most important combinations of gestures, anatomic location, and surgical function for the ML prediction of these outcomes. These results suggest that adding anatomic and functional context strengthens the utility of surgical gestures to predict patient outcomes and augments our understanding of these predictions to guide actionable feedback in the future.

In this study we built upon our prior work demonstrating that a sequence of surgical gestures can be used to predict patient-reported outcomes^6^. To improve the predictive ability of our model, we expanded our original cohort of 80 surgical cases through the addition of 3 surgical centers, 5 surgeons, and 67 patients. We also sought to expand and better describe surgical gestures, the most basic discrete actions during surgery. If gestures are the “what” of surgery, we aimed to add information on “where” and “why” by annotating the anatomic location and surgical task associated with each gesture. This is the first attempt to contextualize gestures to improve their ability to predict surgical outcomes and explain those predictions to extract meaningful insights from that analysis.

Our findings indicate that gesture context adds valuable information to surgical gestures when creating measures of surgical performance. Here we demonstrated that the addition of anatomic and functional context to surgical gestures improves ML predictions of post-operative outcomes compared to gestures alone. The addition of further information to surgical gestures is not only a valid approach, but one that is likely to continue and expand in the future. The sequence of surgical gestures (describing action) alone, while valuable, does not tell the full story of surgery. In order to use gestures as a platform for surgeon feedback, how gestures affect the patient’s tissue must be better understood. Aside from anatomic and functional information, another approach to add context has been to evaluate the *efficacy* of the intended gesture^8^. In other words, if the gesture had the surgeon’s intended effect on the tissue. We previously found that gesture efficacy was closely linked to surgeon experience while others have described the relationship between efficacy and surgeon skills^5,8,9^.

The use of ML models to identify critical parts of surgery has also been used by other groups. ML models have used video and image data to identify the critical view of safety and dangerous dissection zones during laparoscopic cholecystectomy^10,11^. Others have trained AI models on laparoscopic cholecystectomy videos to identify surgical phases^12,13^. ML models have even been used to recognize specific surgical gestures and evaluate surgical skills^14–16^. While identification is important, we aim to determine which events lead to better patient outcomes. To this end, multiple groups have developed ML models using preoperative patient and surgical characteristics to predict surgical complexity and post-operative outcomes^17–19^. ML models are the ideal tools to predict outcomes from thousands of datapoints. These models can also help us identify which of these datapoints is most important. In this study, we apply this approach to the most granular aspect of surgery. To our knowledge, this is the first study to identify gestures with anatomy and functional information most associated with ML predictions of surgical outcomes.

In order to provide actionable feedback to surgeons, we must first understand the causal associations between surgical performance and outcomes. While we are far from asserting *causality*, we will continue to work towards a better comprehension of how the events of surgery are associated with outcomes. We previously linked gesture sequences to surgical outcomes through ML^6^, but these efforts have lacked the ability to explain *why*. In this study, we identified specific combinations of gestures, anatomy, and function that contribute most to our transformer model’s prediction of EF outcomes after RARP.

We found that the gestures of *cold* cut and *spread* in combination during *release NVB* at the *lateral fascia* was among the most important contributors to ML prediction of EF recovery. This specific combination occurred 4.3 times more frequently in the cases of patients who recovered EF after RARP. The other most important sequential CGs were *peel/push* with *cold cut* and *retraction* with *camera move* during *extension* of the *posterior plane*. These results are in line with our prior work finding an increasing probability of EF recovery with more *peel/push* and *spread* gestures^7^. While *cold cut* was not associated with EF outcomes in that prior study, the results presented here indicate that *cold cut* is an important supporting gesture for delicate, blunt tissue dissection alongside *peel/push* and *spread* gestures, often being used to cut the developing lines of tension.

The importance of retraction during the nerve-sparing step is also well documented as minimal retraction is critical to prevent injury and neuropraxia. In a retrospective study of 610 patients, those who had no traction on the NVB during the RARP nerve-sparing step had significantly better EF at 5 months post-op than those who did^20^. Evaluation of traction and other forms of trauma to the NVB are considered essential to the assessment of surgeons in training^21^. Most recently, our group found that retraction gestures directly grasping the NVB were associated with lower likelihood of EF recovery^7^. In this present study, the contribution of *retraction* and *camera move* gesture combinations to predictions of both EF recovery and non-recovery contributes to the growing body of evidence that proper retraction technique during the nerve-sparing dissection is an important contributor to EF recovery.

Unlike gestures such as *retraction, peel/push*, and *spread*, the contributions of *assistant* gestures to EF *non*-recovery have not been described before and represent a novel surgical action associated with EF non-recovery after RARP. Two of the most important gesture combinations for EF non-recovery in this study were *assistant* with *camera move* and *retraction* with *assistant*. This analysis highlights the important role the bedside surgical assistant plays during RARP. Although the specific actions of the assistant were not coded in this study, the *assistant* gesture was often used to improve visualization through either additional tissue retraction or suctioning of blood in the dissection field. This suggests that tissue visualization during dissection and the potential for assistant damage to tissues through retraction or other uncontrolled maneuvers may contribute to EF outcomes and warrant further study. Critically, *assistant* gestures are surgical actions are outside the primary surgeon’s control, yet they may impact surgical outcomes nonetheless. While these findings are informative, the field of model explainability remains in its infancy and determinations of causality remain far-off. The results of this study must be understood as an early attempt to identify specific surgical actions related to post-operative outcomes. We hope that further research and development will allow us to better interpret these associations and, in the future, provide actional feedback to surgeons.

This study has four noteworthy limitations. First, the size of our cohort (147 patients) is relatively small, potentially limiting the predictive capacity of our models. To improve this, we are continuing to prospectively recruit patients undergoing RARP. Building on our previous work, this present cohort already has more patients, surgeons, and surgical centers represented, all of which will continue to increase as we expand our study to more hospitals^6^. Notably, the current process of gesture annotation is resource intensive, requiring approximately 90 minutes of human effort per video^7^. Second, the single surgical procedure used in this study may be viewed as a limitation; however, this methodology of sequencing gestures and providing context around them can be easily applied to additional surgical procedures beyond the prostatectomy. Third, surgical complexity and anatomic differences between patients likely impacts surgical performance and outcomes; however, this is difficult to completely measure and control for. Commonly available datapoints for patient factors such as BMI and prostate size were accounted for in our model, but do not describe the subtle differences in patient anatomy that can influence an operation. Finally, while our contextualized gestures model improves the explainability of EF prediction after RARP, it falls short of providing fully actionable feedback. The findings presented here highlight important gestures for ML predictions of surgical outcomes, but the causal impact of the gestures on outcomes cannot readily be assessed at this time. Critical additional information may include the effect of gestures on the tissue and the intention of the surgeon when using the gesture. Future studies should seek to improve the explainability of model predictions and establish causal relationships between gestures and surgical outcomes.

In this study, we demonstrated that the addition of anatomic and functional context to the sequence of surgical gestures improves ML prediction of EF outcomes after RARP. Using aggregated attention weights, we identified the combinations of gesture, anatomy, and function most strongly associated with EF outcomes. This work represents a proof of principle to identify the key gestures of the nerve-sparing step with the goal of providing immediate, actionable feedback to surgeons in the future.

## Methods

### Study cohort and design

Institutional review board approval was obtained at each participating clinical site. Men undergoing RARP for clinically localized prostate cancer were prospectively evaluated for study inclusion from July 2016 to January 2023. All patients provided informed consent. Inclusion criteria included patients who underwent traditional transperitoneal, bilateral or partial bilateral nerve-sparing technique, had complete surgical video available, follow-up ≥ one year, and intact preoperative EF. The primary outcome was the presence of intact EF one year after surgery. Intact EF was defined as a total score of ≥17 or ≥4 on question 2 of the Sexual Health Inventory for Men (SHIM), a validated and commonly used tool to measure erectile function for sexual intercourse^22^.

### Video annotation

Videos of the bilateral nerve-sparing step from each patient’s surgery were manually annotated for surgical gestures by one of 4 trained raters. Each discrete surgical movement was assigned a surgical gesture according to our previously described validated classification system (Fig. 1)^6^. Small groups of sequential gestures were bundled together according to their anatomic location and the function of the surgical task being performed. All bedside assistant actions utilizing a laparoscopic instrument (e.g. suctioning with suction tip) were categorized as an assistant move without further specification. The raters (JH (resident), RM (resident), UG (resident), CY (student)) underwent standardized annotation training and iterative review until inter-rater agreement of at least 0.80 was achieved. Thereafter, each of the nerve-sparing videos was then annotated by one rater.

### Statistical analysis

Patient characteristics were compared by recovery of erectile function at one year using Wilcoxon rank sum test, chi-square test, an exact test for postoperative Gleason score (for aggressiveness of cancer). As surgeon caseload was based on surgeons’ self-reported estimates of experience prior to this study, and surgeons could contribute multiple surgeries in this analysis when analyzing by caseload, we used mixed effects modeling including a random intercept for surgeon. More specifically, we used univariable mixed effects linear regression to test the association between caseload and features of interest.

### Machine learning model construction

Gesture sequences (i.e., all gestures from the nerve-sparing step in the order of time) and clinical features (i.e., all variables shown in Supplemental Table 1) were used to construct prediction models for one year EF recovery after surgery.

We trained a multi-modal prediction algorithm, consisting of two sub-networks used to handle the entire gesture sequences (a transformer encoder-based network) and clinical features (a fully connected network for the clinical features). The transformer encoder network was used with the self-attention block to leverage the scaled dot-product attention (SDPA) mechanism. This allows the model to capture long-term dependencies by using attention weights to scale the output based on the weighted sum of the encoded gesture representation features, enabling it to focus on the most relevant parts of the entire dissection sequence. Only the transformer encoder network was trained in the case of gesture sequences, gesture sequences with function, gesture sequences with anatomical location, and gesture sequences with function and anatomical location. The input features were represented as d-dimensional vectors, where d denotes number of dependencies considered (e.g., d = 1 for gesture sequences, d = 3 for gesture sequences with functional and anatomical location). A learnable token (CLS) was added to capture the overall dependencies of the sequence features to the token, aiding in explainability of the input sequences with respect to the EF outcome prediction. A classifier head (fully connected network) was used with the input as the encoded token and the output as the binary score for EF outcome positive or negative. A fully connected network was used to predict binary EF outcomes in the case of clinical features. In the case of combined sequences and clinical features input, we fused the encoded representation from the token in the transformer encoder model and the hidden representation in the fully connected network through concatenation and the fused representation was further fed to a fully connected layer with the binary EF outcomes as its output.

The networks were trained using Adam with decoupled weight decay (AdamW) until convergence. The model was then evaluated by a Monte-Carlo cross validation method with a total of 5000 iterations. We first split the total data as 80% train + validation data (118 cases) and 20% test data (29 cases). In each iteration of the cross-validation split, we randomly selected 94 cases as the training data and the remaining 24 cases as the hold-out set to independently validate the model performance. We report the area-under-the-ROC-curve (AUC) and 95% confidence interval (CI) of the test data across the 5000 iterations.

We elected to use a transformer model for its improved explainability characteristics^23^. To demonstrate the feasibility of a transformer model for this purpose, we compared its predictive performance to the LSTM model used in our prior work^6^. Using the inputs of gesture sequence and clinical features (prostate volume, patient age, body mass index, post-op Gleason score, PSA, and prior surgeon caseload) the transformer model achieved an Area Under the Curve (AUC) of 0.80 (95% CI: 0.65, 0.93) for the endpoint of one year erectile function (Supplemental Table 3). Application of the LSTM model utilized in our prior work, but now applied to this dataset, yielded an AUC of 0.74 (95%CI: 0.62, 0.84).

### Explainability analysis

To investigate the explainability of CGs (gesture + function + anatomy) and their relationship to predicting one-year EF recovery versus non-recovery, we leveraged the attention matrices from the transformer architecture to capture the weight each temporal feature received relative to other co-occurring features within each surgical case. To incorporate statistical rigor, we employed a bag-of-words approach to capture relevant CG pair combinations (Fig. 3). We then computed the weighted frequencies of these features for EF recovery and non-recovery cases and assessed their statistical significance using p-values based on differences in weighted frequencies. The three clinically relevant and statistically significant bag-of-words features identified through this analysis are presented in the table.

## Supplementary information

Supplementary information

## Data Availability

The datasets generated and/or analyzed during the current study are not publicly available due to confidential patient information, but are available from the corresponding author on reasonable request.

## Code availability

The underlying code for this is not publicly available but may be made available to qualified researchers on reasonable request from the corresponding author.
