# Supplementary material for: AI model predicts patient outcomes from surgical gestures and provides insights into explainability
Source: NPJ Digit Surg. 2026 May 4;1(1):4. doi: 10.1038/s44484-025-00006-y (PMC13174042; doi:10.1038/s44484-025-00006-y)
Supplement: Supplementary file 1 — Supplementary information [file 44484_2025_6_MOESM1_ESM.pdf]

Supplemental Table 1: Characteristics of patients by recovery of erectile function at 1 year. Values are presented as Median (quartile 1, quartile 3) and frequency (%). P-values were calculated using Wilcoxon rank sum test, chi-square test, an exact test for postoperative Gleason score and logistic regression with a random intercept for surgeon for surgeon caseload.

**Characteristic**	**Not Recovered**, N = 96	**Recovered**, N = 51	**p-value**
Age, year	66 (61, 70)	60 (57, 65)	<0.001
Body Mass Index, kg/m2	28 (26, 30)	28 (25, 30)	0.3
American Society of Anesthesiologists Classification 3 (vs 1-2)	25 (26%)	15 (29%)	0.7
Unknown	1	0	
Preoperative Sexual Health Inventory for Men Score, measuring erectile function	22 (19, 25)	24 (22, 25)	0.005
Unknown	13	2	
Prostate Specific Antigen level (ng/mL)	6.8 (5.4, 10.1)	7.3 (4.9, 9.6)	0.8
Prostate Volume, mL	46 (36, 55)	36 (32, 51)	0.013
Postoperative Gleason Score			0.025
6	5 (5.2%)	8 (16%)	
7	74 (77%)	39 (78%)	
>=8	17 (18%)	3 (6.0%)	
Unknown	0	1	
Full Nerve Sparing	73 (76%)	45 (88%)	0.077
Caseload of surgeon (cases performed total)	2,000 (500, 2,000)	2,000 (825, 2,000)	0.7

Supplemental Table 2. Number and proportion of gestures each function and anatomic location per case in nerve-sparing step. Data presented as Median (Quartile 1, Quartile 3) and each as a proportion of total across all cases.

Feature	Median (Q1, Q3)	Proportion of Total (%)
Total Gestures	600 (435, 967)	100
Peel/push	219 (111, 442)	36
Cold cut	103 (58, 143)	17
Spread	22 (10, 47)	4.5
Hook	12 (7, 19)	2.3
Energy Cut	5 (1, 18)	1.9
Two-hand spread	5 (2, 9)	0.89
Pedicalize	1 (1, 2)	0.030
Burn Dissect	4 (2, 7)	0.31
Retraction	83 (54, 127)	14
Camera Move	86 (49, 112)	13
Coagulation	13 (8, 28)	3.1
Clip	9 (6, 15)	1.8
Assist	17 (9, 28)	3.6
Other	9 (4, 17)	1.3
All Functions	600 (435, 967)	100
Release NVB	322 (198, 532)	54
Extend Posterior Plane	81 (27, 153)	9.4
Investigate	21 (10, 51)	2.5
Coagulate and Cut	6 (2, 14)	0.45
Clip and Cut	55 (34, 95)	11
Stop Bleeding	14 (8, 24)	2.1
Isolate	85 (52, 154)	15
Cleaning Camera	5 (1, 6)	0.14
Apex Dissection	37 (22, 61)	2.7
Other	13 (7, 30)	2.6
All Anatomic Locations	600 (435, 967)	100
Lateral Fascia	218 (143, 375)	42
Prostatic Pedicle	248 (139, 373)	44
Posterior Plane	94 (38, 164)	11
Apex	39 (26, 64)	5.8
Anterior Plane	2 (2, 65)	0.090

Supplemental Table 3. Comparing LSTM (legacy) and Transformer (current) models

Data	LSTM AUC (95% CI)	Transformer AUC (95% CI)
Gen 1	0.68 [95%CI: 0.50, 0.86]	0.78 [95%CI: 0.60, 0.92]
Gen 1 + Clinical Features	0.74 [95%CI: 0.62, 0.84]	0.80 [95%CI: 0.65, 0.93]

Clinical Features include: prostate volume, age, body mass index, post-op Gleason score, PSA, prior surgeon caseload
